# Safe mass drug administration and trachoma elimination

**Published:** 2019-09-10

**Authors:** David Addiss, Virginia Sarah, Nebiyu Negussu, Paul Emerson

**Affiliations:** 1 Director: Focus Area for Compassion and Ethics (FACE), Task Force for Global Health, Decatur, GA, USA.; 2 Global Partnerships Executive, The Fred Hollows Foundation, London, UK; 3 Neglected Tropical Diseases Team Leader: Federal Ministry of Health, Addis Ababa, Ethiopia.; 4 Director, International Trachoma Initiative, Task Force for Global Health, Decatur, GA, USA.

**Simple measures and new Zithromax® dosing guidelines protect children and ensure the safety of mass drug administration.**

**Figure:**
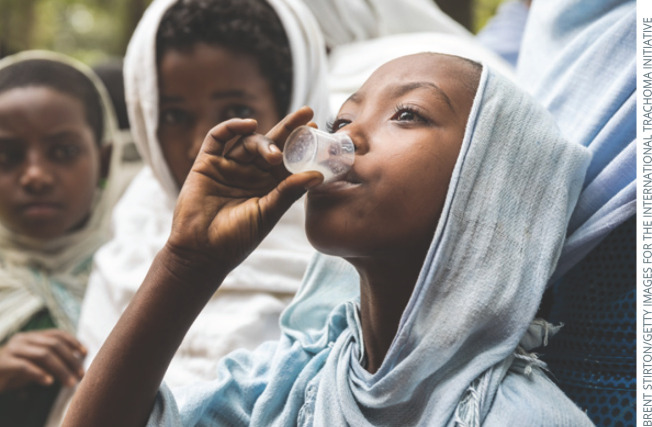
Embet Belachew, aged 7, receiving Zithromax® in the form of oral suspension in the North Mecha Woreda, Amhara Region. ETHIOPIA

Mass drug administration (MDA), which involves giving medicines to a whole community at one time, usually once a year, is a major component of the SAFE strategy to eliminate trachoma (Surgery, Antibiotics, Facial cleanliness, and Environmental improvement) and a cornerstone of programmes to eliminate other neglected tropical diseases (NTDs).

For the past decade, NTD programmes have focused on increasing the number of people treated. Since 2016, more than 1 billion people a year receive treatment through MDA for the NTDs that affect their communities. However, global health programmes have an obligation not only to provide benefits to populations, but also to minimise harm to individuals.

Although MDA medicines are pharmacologically safe, young children have died after choking on tablets for NTDs. The available, but limited, evidence suggests that forcing children to swallow tablets against their will is the main risk factor.

Choking during MDA is a medical error (in this instance, a failure to administer the drug as it is intended) that leads to a preventable adverse event (death due to choking). Medical errors have clearly defined risk factors, such as fatigue, crowding, poor communication, being rushed, and task overload^1^ – all of which can occur in MDA settings. The risk of such events can be reduced through:

- Advanced planning and safety training that emphasises role-playing, problem-solving, communication, orderly work flow, and resilience
- Suitable guidelines and protocols
- Supportive supervision, as outlined in the International Coalition for Trachoma Control's manual for training supervisors.^2^

In trachoma programmes, choking-related deaths can be largely avoided because azithromycin can be given as a powder for oral suspension (POS). When reconstituted with water, this becomes a sweet-tasting syrup.

To promote MDA safety, the International Trachoma Initiative have recently made adjustments to MDA protocols, including:

- Increasing the upper age recommended for POS to 7 years and the minimum height for tablets to 120 cm
- Removing the one- and two-tablet doses from dosing poles to simplify dosing and accommodate the expanded use of POS
- Reiterating the recommendation to offer POS to anyone, of any age, who has trouble swallowing tablets.

MDA safety ultimately depends on the quality of the interaction between the community drug distributor (CDD) and the person taking the medicine (or in the case of young children, the child's parent or guardian). CDDs must follow treatment guidelines and be adequately trained, prepared, and able to effectively communicate with parents and children. Current recommendations for CDDs, reflected in the ITI Zithromax® Management Guide 2019^3^ are as follows:

- Adhere to the new dosing guidelines for trachoma: give POS to children aged 7 or younger and offer tablets only to people who are 120 cm or more in height
- Offer POS to anyone, of any age, who has trouble swallowing tablets
- Directly observe all treatments
- Never force children to take azithromycin, hold their nose to make them swallow, or force their head back to give them the medicine – this increases the risk of choking
- For children who are fussy, irritable, or resist taking azithromycin, reassure the parent or guardian and give them time to calm the child, so the child can receive the treatment
- If the child continues to resist taking azithromycin, do not treat the child during this round of MDA.

Observational assessments of MDAs are needed to evaluate current safety practices and to identify prevention strategies. Prompt investigation, management and reporting of serious adverse events are not only legal and regulatory requirements; they also serve to decrease rumours, restore trust, and sustain high MDA coverage.

The push to reach the high coverage required for trachoma elimination need not conflict with MDA safety – high-quality programmes can achieve both.
